# The alkylation response protein AidB is localized at the new poles and constriction sites in *Brucella abortus*

**DOI:** 10.1186/1471-2180-11-257

**Published:** 2011-11-23

**Authors:** Delphine Dotreppe, Caroline Mullier, Jean-Jacques Letesson, Xavier De Bolle

**Affiliations:** 1 URBM, University of Namur (FUNDP), 61 rue de Bruxelles, Namur, 5000, Belgium

## Abstract

**Background:**

*Brucella abortus *is the etiological agent of a worldwide zoonosis called brucellosis. This alpha-proteobacterium is dividing asymmetrically, and PdhS, an essential histidine kinase, was reported to be an old pole marker.

**Results:**

We were interested to identify functions that could be recruited to bacterial poles. The *Brucella *ORFeome, a collection of cloned predicted coding sequences, was placed in fusion with yellow fluorescent protein (YFP) coding sequence and screened for polar localizations in *B. abortus*. We report that AidB-YFP was systematically localized to the new poles and at constrictions sites in *B. abortus*, either in culture or inside infected HeLa cells or RAW264.7 macrophages. AidB is an acyl-CoA dehydrogenase (ACAD) homolog, similar to *E. coli *AidB, an enzyme putatively involved in destroying alkylating agents. Accordingly, a *B. abortus aidB *mutant is more sensitive than the wild-type strain to the lethality induced by methanesulphonic acid ethyl ester (EMS). The exposure to EMS led to a very low frequency of constriction events, suggesting that cell cycle is blocked during alkylation damage. The localization of AidB-YFP at the new poles and at constriction sites seems to be specific for this ACAD homolog since two other ACAD homologs fused to YFP did not show specific localization. The overexpression of *aidB*, but not the two other ACAD coding sequences, leads to multiple morphological defects.

**Conclusions:**

Data reported here suggest that AidB is a marker of new poles and constriction sites, that could be considered as sites of preparation of new poles in the sibling cells originating from cell division. The possible role of AidB in the generation or the function of new poles needs further investigation.

## Background

*Brucella *is the etiologic agent of brucellosis, a worldwide zoonosis that affects a broad range of mammals, including humans [1]. *Brucella *is considered as a facultative intracellular pathogen that enters various cell types during the infection process, including macrophages and epithelial cells, and ultimately survives and multiplies inside these cells [2]. After internalization, intracellular *Brucella *resides within a vacuole (BCV for *Brucella*-containing vacuole) that interacts with early endosomes [3] and then transiently acquire markers of late endosomes such as LAMP1. In epithelial cells and macrophages, non-opsonized bacteria replicate finally in a compartment characterized by the presence of endoplasmic reticulum (ER) markers [4-7].

The mechanisms used by *Brucella *to sense and respond to changes within the intracellular environment are poorly understood. *Brucella *spp. seem well adapted to cope with nutritional [8] and various physicochemical stresses encountered in non-professional and especially professional phagocytes [9]. For example, *Brucella *spp. are adapted to oxidative and nitrosative stresses [9] that have been shown to affect genome integrity in other bacterial species. In 2002, Köhler *et al*. identified an attenuated mutant with a mini-transposon in the *aidB *gene, proposed to encode an acyl-CoA dehydrogenase homolog [10]. In *Escherichia coli*, AidB protein takes part of the adaptative response to alkylating agents that could damage the genome [11], suggesting that AidB homolog could play a similar role in *B. abortus*. Moreover, a *Brucella melitensis *mutant in the *alkA *gene was found to be attenuated in mice (Pascal Lestrate, Ph.D. thesis, 2003). The *alkA *gene is homologous to *E. coli alkA*, another gene involved in the adaptative response to alkylating stress [12,13]. In summary, these data suggests that DNA alkylation repair systems could play a role in intracellular persistence, possibly by preventing DNA damage that might be induced by alkylating agents, either produced from endogenous sources [14] or induced by the host during the infection process.

Here we report that while screening *Brucella *ORFeome for polar proteins in *Brucella abortus*, AidB was found to localize at the new pole, as well as at the constriction site in dividing cells. This pattern of localization is maintained in *B. abortus *infecting epithelial cells and macrophages at different times post-infection. Analysis of an *aidB *mutant revealed on one hand no effect on virulence, and on the other hand that the *aidB *mutant was more sensitive to the alkylating agent methanesulfonic acid ethyl ester (EMS), suggesting a function of AidB in the defence against DNA methylation damage. While EMS was found to block cell cycle before cell constriction, a *B. abortus *strain overexpressing *aidB *was found to generate multipolar morphologies, suggesting a link between the response to alkylating agents and cell growth and/or division.

## Results

### Screen for polarly localized proteins in *Brucella abortus*

To identify polar proteins at the genomic scale, we took advantage of the *Brucella melitensis *ORFeome [15], a collection of all predicted coding sequences (pCDSs) from *B. melitensis *genome cloned in a donor vector (pDONR201) allowing the Gateway recombinational cloning. The resulting ~3200 entry clones are physically organized in 96-well plates (34 plates), each well containing one entry clone (one cloned *B. melitensis *pCDS). For some large-scale experiments, the *Brucella *ORFeome is also organized in 68 pools [16], each pool being a mix of clones from one half-plate of the original ORFeome. Each of the 68 pools was used to transfer the pCDSs in a destination vector allowing pCDS-*yfp *fusions under the control of *E. coli lac *promoter, on a low copy number plasmid. More than 1000 transformants (> 10 times the diversity of the pool) were generated for each cloned pool of pCDSs. The pools of constructions were transformed into *E. coli *strain S17-1 (> 1000 transformants/pool) and were transferred in a *Brucella abortus *XDB1155 strain [16] by mating. The XDB1155 strain produces the PdhS-CFP (cyan fluorescent protein) fusion protein from the chromosomal *pdhS *locus. This strain allows the quick determination of the nature of the pole marked by the protein-YFP fusion since PdhS-CFP is known to specifically label the old pole [17]. The diversity of the pCDSs in the pools was checked by PCR and restriction analysis on isolated clones from 5 different pools with various average pCDSs sizes, in *E. coli *S17-1 and *B. abortus *XDB1155 strains. The analysis of restriction profiles suggests that there is no main over-representation of a given clone in the examined pools.

For the screening strategy, we observed the 68 pools using fluorescence microscopy, and we selected pools in which a fraction of the clones exhibit a polar YFP fusion. The pooled clones were examined after cultivation on solid medium and > 1000 bacteria were observed on agarose pads. Afterwards, pools bearing polar localization were observed clone by clone in the same way to identify clones producing polar proteins. The pCDS allowing polar localization were amplified by PCR and sequenced to allow their identification.

Before analysing the 68 pools, we first screened a pool supposed to contain the *pdhS *coding sequence (CDS), as a positive control. The complete procedure was applied and six clones were identified as polarly localized, and all of them contained the *pdhS *CDS fused to YFP. This pilot study suggested that the screening procedure was working, and that PdhS was the main polar protein in this pool. The analysis of the 67 remaining pools led to the selection of 8 pools for which a significant proportion of bacteria showed polar foci. The average size of the pCDSs contained in the 8 pools was heterogeneous, varying from 450 to 2000 bp. In one of these 8 pools, we identified a pCDS of interest (BMEII0671 and BAB2_0642 in *B. melitensis *16M and *B. abortus *2308 genomes, respectively), that we named *aidB *by homology with *E. coli aidB*.

### *Brucella *AidB is member of the acyl-CoA dehydrogenase family

Deduced AidB sequence is 551 amino acids long, with a predicted molecular mass of 60 kDa and without predicted transmembrane segments. The AidB sequence is similar to acyl-CoA dehydrogenases (ACADs), proteins generally involved in the fatty acid β-oxidation. In the *B. melitensis *16M genome, eight pCDSs are proposed to encode enzymes similar to ACADs. *B. melitensis *and *B. abortus *AidB deduced sequences are 100% identical. *Brucella *AidB presents 42% identity to the *Escherichia coli *AidB (E value of 4 10^-117 ^when *B. abortus *AidB deduced sequence is blasted against *E. coli *genomes), suggesting a functional conservation between these enzymes. This is further supported (1) by the conservation of the Glu-Gly catalytic pair at positions 425 and 426 (433 and 434 in *Brucella *AidB), (2) by the conservation of 9 of the 10 residues involved in FAD binding, the non-conserved residue being a Ser residue replaced by a Thr at position 198 of *B. abortus *AidB, and (3) the similarity of the regions involved in the formation of the tetrameric structure of *E. coli *AidB (10 residues identical on 19 residues). Moreover, a specific feature of *E. coli *AidB, compared to other members of the ACADs family, is the presence of a Trp424 residue, involved in the shaping of the substrate-binding pocket. This residue is conserved in *B. abortus *AidB (Trp432). Altogether, these data suggest that *B. abortus *AidB could play a similar role as *E. coli *AidB, except that the region of *E. coli *AidB involved in DNA binding (about 100 C-terminal residues, Additional file 1 for sequence alignment and Additional file 2 for three-dimensional model), is not conserved in *B. abortus *AidB. This suggests that *B. abortus *AidB could be unable to bind DNA, or would bind a very different sequence. Indeed, in *E. coli *AidB is a multifunctional protein proposed to be involved in the destruction of alkylating agents before they reach DNA [18] and in the transcriptional control of the *aidB *promoter [19]. It is thus possible that only the enzymatic activity of AidB is conserved in *B. abortus*, and not its ability to bind a specific DNA sequence in the *aidB *promoter. In *E. coli*, exposition to alkylating agents stimulates expression of *aidB, ada, alkA *and *alkB *genes [20], Ada, AlkA and AlkB proteins being actively involved in the repair of alkylated DNA [21]. Ada, AlkA and AlkB homologs are found in the *Brucella *genomes (data not shown), suggesting that these bacteria are able to resist to an alkylation stress.

### The *aidB *mutation leads to increased sensitivity to the DNA-alkylating agent EMS

**Figure 1:**
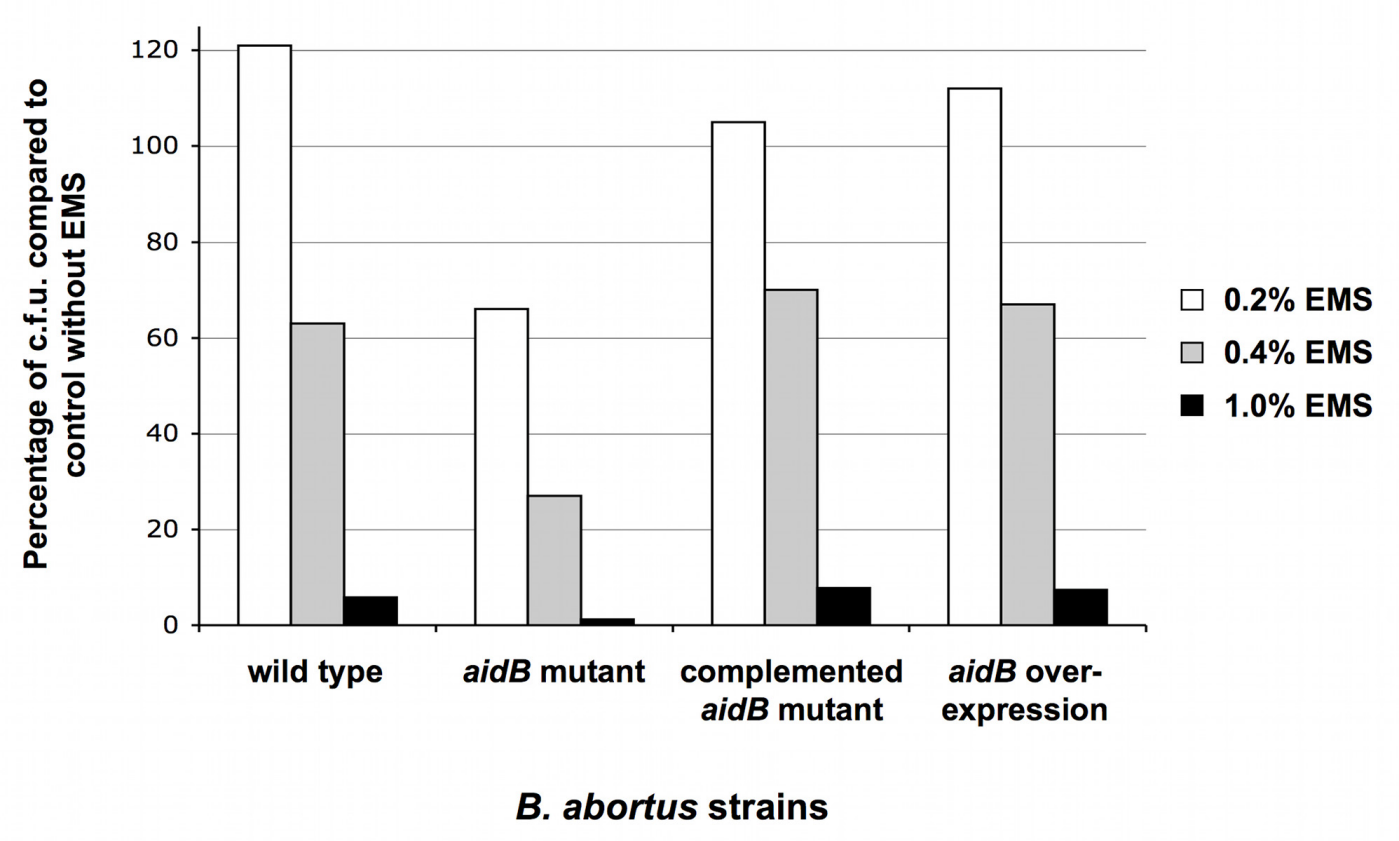
**The *B. abortus aidB *mutant is more sensitive to EMS**. The sensitivity of *B. abortus *wild-type, *aidB *mutant strain, complemented *aidB *mutant and *aidB *overexpression strains was scored by counting the c.f.u. recovered after 4 h of incubation 2YT medium at 37°C, in the presence of 0.2, 0.4 or 1% EMS. The results are expressed as the percentage of c.f.u. compared to a control in which EMS was omitted. Bacteria were obtained from cultures stopped during exponential growth phase.

To investigate the putative function of *B. abortus *AidB protein, we tested the effect of the *aidB *mutation on the survival during an alkylating stress. A *B. abortus *544 strain with a disrupted *aidB *gene was constructed (XDB1121 strain). An *aidB *overexpression strain was constructed by inserting a medium-copy plasmid (pDD003) bearing the *aidB *coding sequence in *B. abortus*, generating the XDB1122 strain. The disruption and overexpression strains (XDB1121 and XDB1122, respectively) were analyzed for their sensitivity to the alkylating agent EMS. In summary, the parental strain, the disruption strain (XDB1121), the overexpression strain (XDB1122) and the complemented strain (XDB1127) were incubated in 2YT medium with 0.2, 0.4 and 1.0% EMS for 4 h at 37°C. The alkylating agent was then removed, and serial dilutions of the cultures were plated on 2YT agar. The number of colony forming units (c.f.u.) was determined and the percentage of survival after treatment was expressed by comparison to a culture of these different strains without EMS. A representative result is shown in Figure 1. After exposure to EMS (0.2 to 1%), the disruption strain (XDB1121) was more sensitive than the parental strain and the disruption strain complemented with a low copy plasmid carrying the *aidB-yfp *fusion (XDB1127). This data was confirmed by reconstructing three independent *B. abortus aidB *mutants that were more sensitive than the wild-type strain to the presence of 0.4% EMS for 4 h. Indeed, we observed 10.2% ± 2.0 survival for the *aidB *mutants (n = 3), compared to 62% survival for the wild-type strain. This phenotype was complemented for the three strains, since we observed 61.3% ± 9.1 survival after 4 h in 0.4% EMS for the three *aidB *mutants complemented with the pDD001 plasmid (Table 1). In order to confirm that *aidB *mutant was more sensitive to alkylating agents and not just EMS, we also tested the sensitivity of the *aidB *mutant and wild type strain to methyl methanesulfonate (MMS), another alkylating agent. After 4 h of incubation with 0.02% MMS in rich medium, 45% of survival was obtained for the wild type strain, while only 2.1% of the *aidB *mutants survived, according to c.f.u. counting. Altogether, these experiments indicate that the *B. abortus aidB *gene is probably involved in the repair or the prevention of alkylation damage, as suggested by its homology with *E. coli *AidB. It also indicates that AidB remains active when it is fused to YFP.

**Table 1:** Strains and plasmids

Strain	Relevant Genotype or Description	Reference or Source
*B. abortus*		
544 Nal^R^	Nalidixic acid-resistant *B. abortus *544	J-M. Verger
XDB1155	*B. abortus *544 *pdhS-cfp*	[16]
XDB1120	XDB1155 + pDD001	This study
XDB1121	Disrupted *aidB *in *B. abortus *544 Nal^R^	This study
XDB1122	XDB1155 + pDD003	This study
XDB1123	XDB1155 + pDD007	This study
XDB1124	XDB1155 + pDD008	This study
XDB1127	XDB1121 + pDD001	This study
XDB1118	*B. abortus *544 with integrated pCVDH07	This study and [33]
XDB1128	XDB1118 + pDD001	This study
*E. coli*		
DH10B	Cloning strain	Invitrogen
S17-1	RP4-2, Tc::Mu,Km-Tn7, for plasmid mobilization	[26]

**Plasmid**	**Relevant Genotype or Description**	**Reference or Source**

pDONR201	BP cloning vector	Invitrogen
pRH005	Gateway-compatible YFP low copy vector	[34]
pRH016	Gateway-compatible pBBR1-MCS1-3HA	[34]
pDD001	pRH005 carrying *aidB*	This study
pDD002	pDONR201 carrying *aidB*	This study
pDD003	pRH016 carrying *aidB*	This study
pDD007	pRH016 carrying *acaD1*	This study
pDD008	pRH016 carrying *acaD2*	This study

### AidB-YFP is localized at the new pole, and at the constriction site in dividing cells

**Figure 2:**
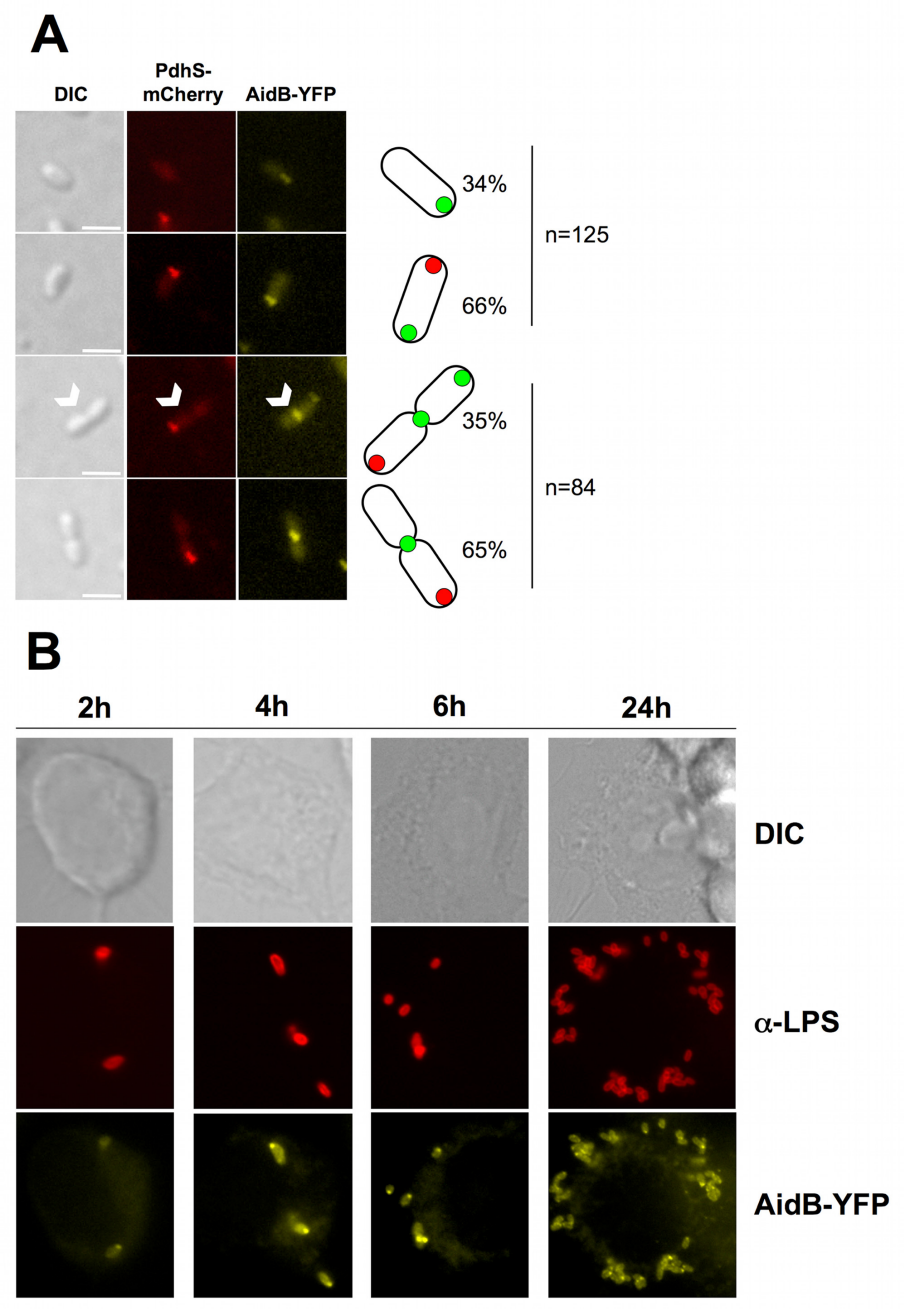
**The *B. abortus *AidB-YFP is localized at new poles and at constriction sites, in culture and in macrophages**. The *B. abortus *XDB1128 strain was carrying an *aidB-yfp *fusion on a low copy plasmid, and *pdhS-mCherry *at the *pdhS *chromosomal locus. (A) Bacteria were grown in rich medium and the pictures were taken in exponential phase. Differential interference contrast (DIC) is shown on the left. The white arrowheads indicate the dividing cell in which two AidB-YFP foci are detectable. Each scale bar represents 2 μm. The bacterial types are schematically drawn on the right side of the pictures, as they are represented in figure 6. The two upper panels were made with non-diving bacteria, and counting was made with 125 bacteria. The two lower panels were made with dividing bacteria, and counting was made on 84 dividing bacteria. (B) RAW264.7 macrophages were infected for 2, 4, 6, or 24 h with the *B. abortus *strain expressing *aidB-yfp *(XDB1120). The infected cells were fixed and immunostained with 12G12 anti-lipopolysaccharide ("α-LPS") primary antibody and anti-mouse secondary antibody coupled to Texas Red. A majority of the bacteria present a single focus of AidB-YFP, suggesting that polar localization of this fusion is also occurring during the infection of macrophages.

The localization of the AidB-YFP fusion protein was analyzed in a *B. abortus *strain carrying a chromosomal *pdhS-mCherry *fusion (XDB1128 strain) during the exponential growth phase in liquid cultures, in rich medium (Figure 2A). In these conditions, the localization of the AidB-YFP fusion protein displayed three patterns, depending on the presence or the absence of a constriction site. In bacteria without detectable constriction, AidB-YFP localized at the new pole and PdhS-mCherry at the old pole in 66% of the bacteria (n = 125), with 34% of bacteria labelled only with polar AidB-YFP and not PdhS-mCherry. In the bacteria displaying a constriction site, 65% (n = 84) displayed a single AidB-YFP focus at the constriction site, while the remaining 35% have two foci of AidB-YFP, one at the "young" pole and one at the constriction site. Here we define a "young" pole as a new pole that is becoming old, because bacteria show a detectable constriction, meaning that there is uncertainty about the completion of cytokinesis, and therefore uncertainty about the status of this pole (either new or old). We never observed the PdhS-mCherry and AidB-YFP fusions at the same pole (n = 256) (Figure 2A). Western blots analysis using an anti-GFP antibody on this strain suggested that AidB-YFP fusion was stable when it was produced from the low-copy plasmid pDD001 (data not shown). As proposed in the model depicted in the discussion, the cells labelled with polar AidB-YFP without polar PdhS-mCherry could correspond to bacteria produced by division of cells carrying PdhS-mCherry at the old pole and AidB-YFP at the constriction site. Indeed, after cell division, one of the two cells does not inherit PdhS-mCherry from the mother cell, but AidB-YFP at the constriction site is proposed to be transmitted to the new pole of this daughter cell.

**Figure 3:**
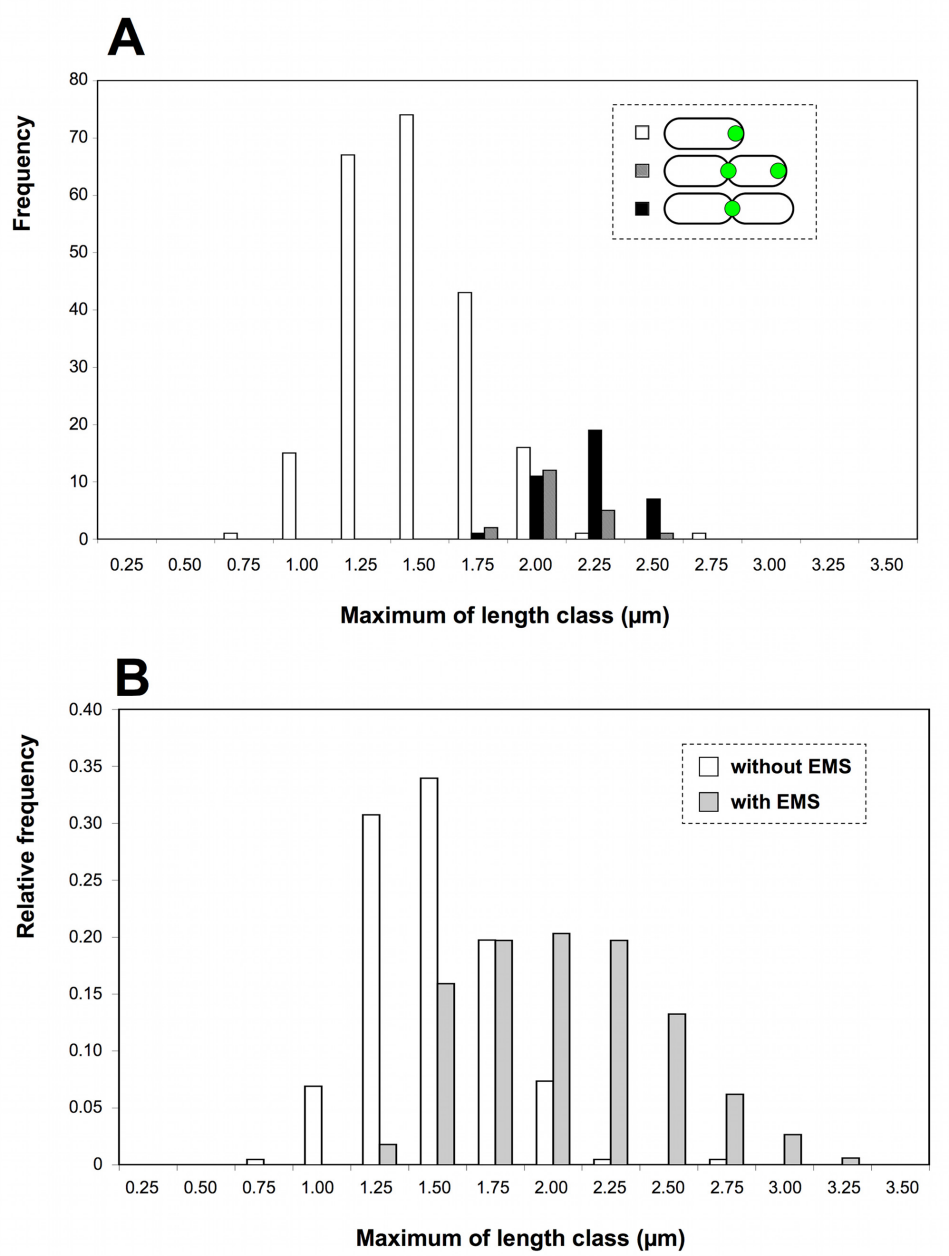
**Size distribution of *B. abortus *carrying AidB-YFP, in the presence or absence of an alkylating agent (EMS)**. The bacterial lengths were grouped in classes of 0.25 μm, and the maximum value for each class is given on the × axis. (A) Size distribution of 276 bacteria (XDB1128 strain) with AidB-YFP either at the new pole (white), the new pole and the constriction site (dark grey), or the constriction site only (black). (B) Size distribution of *B. abortus *(XDB1128 strain) exposed to 0.4% of EMS for 4 h (light grey, n = 340) or the unexposed control (white, n = 218, bacteria without detectable constriction). (C) DIC and fluorescence pictures of the XDB1128 strain expressing *aidB-yfp *and *pdhS-mCherry *fusions, as described in figure 2. The bacteria in the lower panels have been exposed to 0.4% EMS for 4 h in rich (2YT) medium. On the top panels, control bacteria were incubated for 4 h in 2YT in the absence of EMS. Constriction sites are indicated by arrowheads. Each scale bar represents 2 μm.

Interestingly, the cells harbouring the two AidB-YFP foci are significantly (*p *< 0.005) smaller (1.93 μm on average) than the bacteria having a single focus of AidB-YFP at the constriction site (2.08 μm on average), suggesting that in the cell cycle, bacteria with 2 foci precede those with a single focus at the constriction site (Figure 3A). This feature of the cell cycle is depicted in the discussion.

Furthermore, the localization of AidB-YFP is still at the new pole after 4 h of exposure with 0.4% EMS (80% of the bacteria exhibited PdhS-mCherry at one pole and AidB-YFP at the opposite pole, n = 237). This observation indicated that AidB-YFP is not released from the new pole in the presence of an alkylating stress with EMS, further suggesting that AidB is active at the new pole, because in these conditions an *aidB *mutant is killed. Interestingly, bacteria exposed to EMS displayed detectable constriction at the much less frequency (2 constrictions observed among 254 bacteria) compared to the untreated control (44 constrictions observed among 254 bacteria). Moreover, bacteria treated with 0.4% EMS for 4 h and were significantly (*p *< 0.001) longer on average than unconstricted bacteria that were not exposed to EMS (Figure 3B). This suggests that growth is not arrested by the presence of EMS, while constriction is clearly inhibited. This is consistent with a replication arrest caused by alkylation of the bacterial genome, as previously reported for *E. coli *[22].

### AidB polar localization persists inside host cells

*B. abortus *is an intracellular pathogen that encounters various stresses during its life cycle [9]. Since these stresses could result in the alkylation of DNA, e.g. through nitrosative stress [14], we tested the localization pattern of AidB-YFP in *B. abortus *(XDB1120 strain) during an infection of human epithelial cells (HeLa cells). At 6 h and 24 h post-infection, infected cells were fixed, and bacteria were detected using a monoclonal anti-lipopolysaccharide antibody. The polar foci of AidB-YFP were similar to those observed in bacteriological culture, suggesting that in these conditions, there is no systematic delocalization of AidB-YFP. Similar results were also obtained with XDB1120 strain in RAW264.7 macrophage infection. At 2 h, 4 h, 6 h and 24 h post-infection, AidB-YFP fusion proteins were still polar (Figure 2B).

### Morphological analysis of *aidB *disruption and overexpression mutants

**Figure 4:**
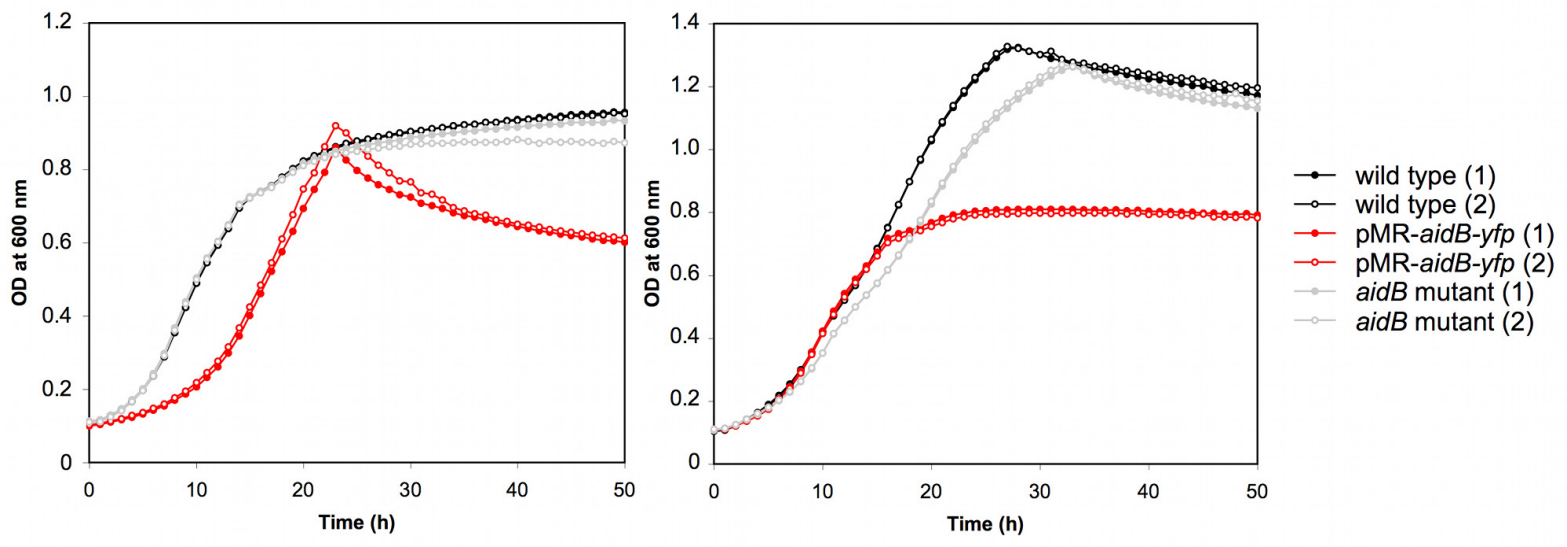
**Growth defect of the *B. abortus *strain expressing the *aidB-yfp *fusion (XDB1120)**. The growth of *B. abortus *wild-type, *aidB *mutant and XDB1120 (pMR-*aidB-yfp*) strains was followed by recording OD at 600 nm in a Bioscreen. Duplicates (1) and (2) are shown for each strain, for 2YT (left panel) or tryptic soy broth (right panel) as culture media. In both culture media, the OD_600 _during stationary culture phase of the XDB1120 strain is lower compared to the wild type control.

**Figure 5:**
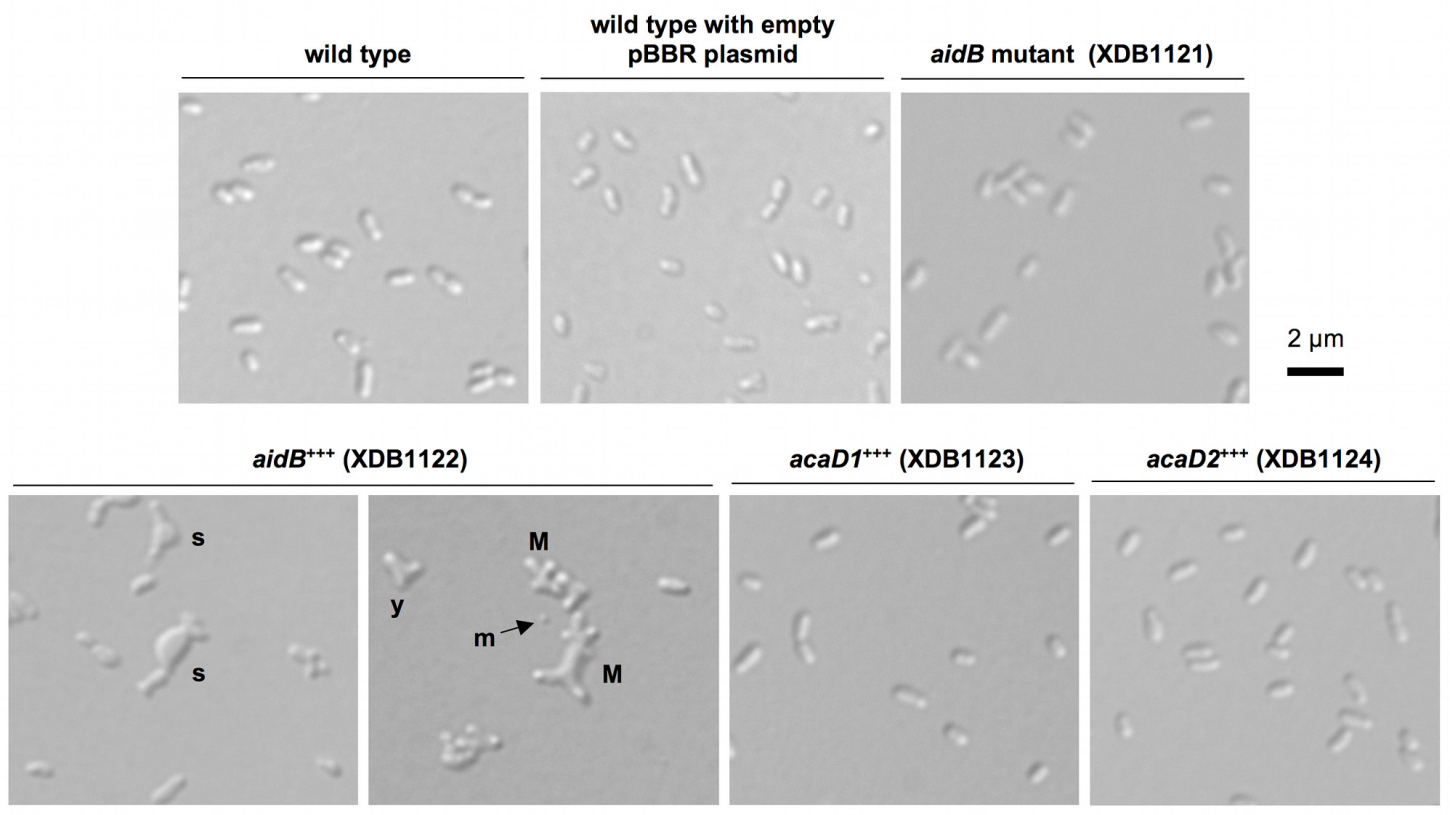
**Morphological defect of the *B. abortus aidB *overexpressing strain**. Differential interference contrast (DIC) images were taken with bacteria of the *aidB *(*aidB*^+++^), *acaD1 *(*acaD1*^+++^) and *acaD2 *(*acaD2*^+++^) overexpression strains, the *aidB *disruption strain, and the wild-type strain with or without the control pBBR1MCS plasmid [32], without insert. Two panels are shown for the *aidB *overexpression strain, the only strain displaying a morphological defect during stationary culture phase. The morphological defects are multiple, with multipolar bacteria (M), Y-shaped cells (y), swollen cells (s) and some minicells (m).

Since AidB-YFP is mainly polar, we tested whether either a disruption or an overexpression of the *aidB *gene affects growth, bacterial morphology, and virulence in cellular models of infection. The growth curve of an *aidB *mutant (XDB1121) strain was similar to the wild-type control in 2YT medium (Figure 4). The *aidB *mutant strain (XDB1121) was morphologically indistinguishable from the wild-type strain (data not shown and Figure 5). The localization AidB-YFP fusion protein (expressed from pDD001) was similar in the *aidB *mutant compared to the wild-type strain (data not shown), suggesting that polar localization of AidB-YFP does not depend on the presence of endogenous AidB, not fused to YFP. The virulence of the *aidB *mutant in HeLa cells and RAW264.7 macrophages was also similar to the wild-type strain (data not shown and Additional file 3). In summary, the *aidB *gene seems to be dispensable for growth in bacteriological medium, maintenance of cell shape and for *B. abortus *virulence in a cellular model of infection.

The growth curve of the strain expressing *aidB-yfp *(XDB1120) in rich media (2YT or tryptic soy broth) is abnormal compared to the wild-type strain (Figure 4). Indeed, the OD during the stationary phase is lower OD with the XDB1120 strain compared to the wild-type control. In stationary phase in 2YT medium, the OD of the *aidB *overexpression strain is even decreasing while the OD of the wild-type control remained stable. This correlates with a higher frequency of dead cells in the *aidB *overexpression strain XDB1122 (22.8% in stationary phase, n = 400) compared to the wild-type strain (5.2% dead cells, n = 400) or the wild-type strain with an empty pBBR1 plasmid (6.7% dead cells, n = 400), the backbone of the *aidB *overexpression plasmid in XDB1122 strain. This observation suggests that *aidB *overexpression is partially lethal in stationary phase. In stationary phase cultures of the XDB1120 strain, the bacteria display abnormal morphologies at much higher frequency (22%; n = 200) than the wild-type strain (< 1%; n = 200). This phenotype is probably due to the overproduction of AidB-YFP because the *aidB *overexpression strain (XDB1122) displayed similar morphological defects (61%; n = 200) (Figure 5). Among these abnormal morphologies, bacteria with multipolar shapes were very frequent, swollen cells were often observed, as well as Y-shaped bacteria, elongated cells and minicells. The morphological phenotype of this strain is thus pleiotropic. The analysis of AidB-YFP and PdhS-CFP localization in XDB1120 bacteria with aberrant morphologies, during the exponential growth phase, did not yield a systematic localization pattern, the AidB-YFP and PdhS-CFP fusions being often diffuse in the bacterium (data not shown).

### Subcellular localization and overproduction effects of AidB are specific to this acyl-CoA dehydrogenase homolog

Since AidB is a member of the 8 ACADs paralogs, we wondered if the particular localization of AidB-YFP and the presence of multipolar forms for the *aidB *overexpression mutant were specific characteristics of this ACAD homolog. We chose two *B. abortus *ACAD homologs that are stably produced at a detectable level using Western blot (data not shown). Both paralogs were annotated (BAB2_0433 and BAB2_0216, respectively named AcaD1 and AcaD2) as ACADs and would be involved in the fatty acid β-oxidation pathway. We observed that both ACADs homologs had a diffuse localization in the cytoplasm when fused to YFP (XDB1123 and XDB1124 strains, data not shown), suggesting that the particular localization of AidB-YFP (at young poles and at the constriction site in dividing cells) is not a common characteristic shared by all ACADs homologs in *B. abortus*. The phenotype of the strains overproducing one of these two ACADs homologs is similar to the *B. abortus pdhS-cfp *control strain (Figure 5), with a very low frequency (< 1%) of morphological defects. This suggests that overexpression of any *ACAD *gene does not produce a morphological defect in *B. abortus*, further supporting a specific -although probably indirect- role of *aidB *in events related to morphogenesis.

## Discussion

**Figure 6:**
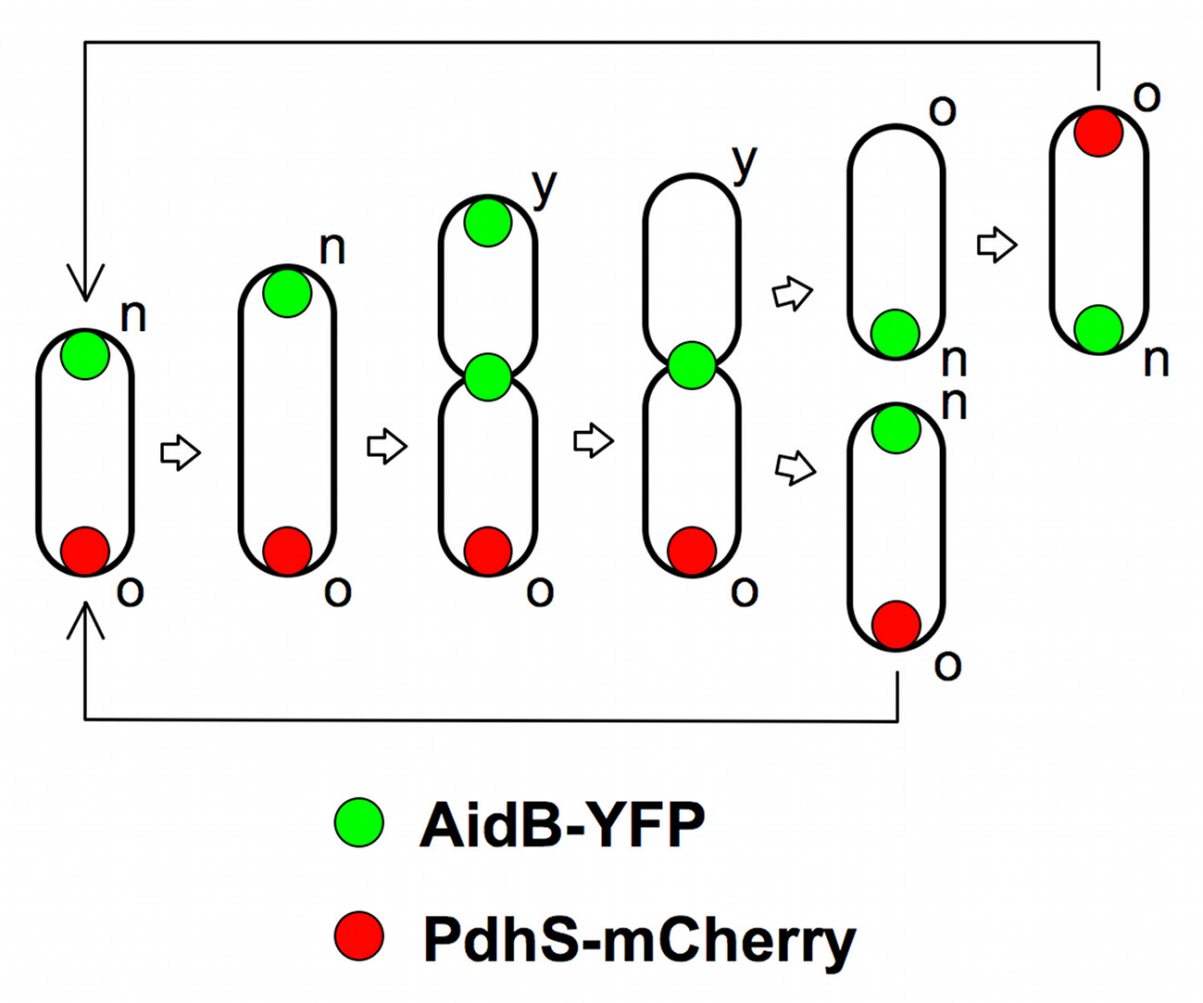
**Model for the localization of AidB-YFP along *B. abortus *cell cycle**. The PdhS-mCherry is labelling the old pole of *B. abortus*. AidB-YFP is therefore localized at the new pole, as suggested by Figure 2. In dividing cells, we hypothesize that AidB-YFP is first present at the young pole (the new pole that becomes old) and at the constriction site. This localization at the young pole would be lost afterwards, allowing the generation of two sibling cells with a unique pole of AidB-YFP. The new (n), young (y) and old (o) poles are labelled. In this model, the constriction region would be the preparation site for the new poles of the sibling cells.

The screen of the ORFeome for polar localization allowed the identification of AidB, that shows a clear localization pattern at new poles as well as at constriction sites and/or at young pole in cells in division in bacteriological medium. The polar localization of AidB-YFP is preserved in HeLa cells and RAW264.7 macrophages at different times post-infection. We therefore propose that AidB is a marker of new poles and constriction sites. To the best of our knowledge, it is the first time that a particular subcellular localization is described for one of the actors involved in the alkylation damage repair. Interestingly, the constriction site corresponds to the location of the future new poles just after completion of cell division. We therefore propose a model (Figure 6) in which AidB-YFP is not only localized at the new pole, but also at the constriction site in dividing cells, a mechanism by which AidB-YFP would be ideally localized for a localization at the new pole in newly formed sibling cells. This model implies that when new poles mature to old poles, after cell division, they are no longer labelled with AidB-YFP (Figure 6).

In the conditions tested, overexpression of *aidB *leads to bacteria with aberrant morphology (Figure 5). This could be due to defects in cell division, cell growth or coordination between both. One hypothesis would be that AidB could indirectly contribute to the generation of new poles, and overexpression of *aidB *would result in the generation of additional new poles, forming bacteria with abnormal morphology, e.g. multipolar shapes (Figure 5). The selective advantage of the polar localization of AidB is unknown, but it could be related to its role in the adaptative response to alkylating agents, suggested here to block cell cycle before cell division (Figure 3B). This would be consistent with a role of AidB in limiting alkylating damage to DNA, which would logically block replication initiation and/or progression.

The *B. abortus *AidB protein has a high level of identity (42%) to *E. coli *AidB, suggesting functional conservation between the two proteins. This prediction is supported by the increased sensitivity of the *B. abortus aidB *mutant strain to the alkylating agent EMS compared to the wild-type control (Figure 1). *Brucella *genomes contain the *ada, alkA *and *alkB *genes necessary for an adaptative response to alkylation damage similar to the one reported for *E. coli *[11]. We propose that one possible function of AidB would be to help in the detoxification of some alkylating agents, like in *E. coli*. These alkylating agents could be found at particular stages of the infection in the natural hosts, or generated by the bacteria themselves [14]. The absence of attenuation of the *aidB *mutant in HeLa cells or in RAW264.7 macrophages suggests that such alkylating agents are not crucial for the control of the number of c.f.u. during infection of these cell lines. Our data do not confirm the previous observation that a transpositional *aidB *mutant was attenuated in THP-1 macrophages [10], unless these specific macrophages have specific features differentiating them from RAW264.7 macrophages for the generation of an alkylating stress. In *Salmonella enterica*, an *aidB *mutant was more sensitive than the wild-type strain to several alkylating agents but presented no effect on the virulence in the mouse model. Indeed, the virulence of a *S. enterica *mutant defective in all genes specifically involved in DNA alkylation damage repair was not affected [23].

Recently, in *C. crescentus*, Radhakrishnan *et al*. reported that KidO, an NAD(P)-binding oxidoreductase homolog with conserved residues in its NAD(P)-binding pocket, acts directly on the FtsZ tubulin [24]. Localization of KidO to the Z-ring is disrupted by mutations in the cofactor-binding pocket that disturb the association with NAD(P), implying that NAD(P) binding is important for the recruitment of KidO to the Z-ring [24]. In this context, it should be interesting to construct a mutated AidB defective for FAD binding and observe the impact of this mutation on the AidB-YFP localization. Finally, the selective advantage of AidB recruitment at the new pole remains to be discovered. One possibility would be that crucial regions of the nucleoid located close to the new pole, such as replication origins, could be more protected from alkylating agents. This would resemble the proposed specific protection of genes by AidB in *E. coli *[25] that would be dependent on subcellular localization of AidB in *B. abortus*. The aberrant morphology of the strain overexpressing *aidB *indicates that either growth or division are affected, which suggest that AidB could be (indirectly) involved in the control of these processes, for example by providing a checkpoint for cell division.

## Conclusion

AidB is induced during alkylation damage response in *E. coli*, however its molecular function is mostly unknown. Here we report that a *B. abortus aidB *mutant is more sensitive to EMS, suggesting that AidB is playing a functional role in the response to alkylation damage. The AidB-YFP fusion is a marker of new poles (Figures 2 and 6). The AidB-YFP fusion is also localized to constriction sites, which could be considered as preparation sites for new poles in dividing cells. AidB molecular function at the new pole is unknown, but it is expected to be active at this site, since its new pole localization is preserved in *B. abortus *exposed to EMS. Our data also suggest that exposure to EMS blocks the cell cycle before the constriction event, presumably before or during genome replication. The characteristic multipolar morphology of the *aidB *overexpression strain suggests that AidB could (indirectly) play a role in growth or cell division of *B. abortus*.

## Methods

### Strains, plasmids and cell growth

All *Brucella *strains used in this study (Table 1) were derived from *B. abortus *544 Nal^R ^(a spontaneous nalidixic acid-resistant mutant of *B. abortus *544 strain), and were routinely cultivated in rich medium 2YT (1% yeast extract, 1.5% tryptone and 0.5% NaCl, with 1.5% agar for solid medium). *E. coli *strains DH10B (Invitrogen Life-Technologies) and S17-1 [26] were cultivated in LB broth (0.5% yeast extract, 1% tryptone, 0.5% NaCl) with streptomycin. Antibiotics were used at the following concentrations when appropriate: nalidixic acid, 25 μg/ml; kanamycin, 20 μg/ml; chloramphenicol, 20 μg/ml. Plasmids were mobilized from *E. coli *strain S17-1 into *B. abortus *as previously described [27]. Growth curves were monitored using a Bioscreen system (Thermo Fisher, ref. 110001-536), allowing continuous monitoring for growth curves in a multiwell format. *B. abortus *liquid cultures in 2YT medium with the appropriate antibiotic were centrifuged, washed once with PBS and diluted to an OD_600 _of 0.1 in 2YT (or tryptic soy broth) to start the culture in the Bioscreen system. Each culture (200 μl per well) was performed at 37°C.

### *Control of the *B. abortus *strain used for the localization screen*

The fact that the XDB1155 strain is viable and does not present any apparent morphological defects or growth delay suggests that the CFP fusion at the C-terminal of PdhS is not affecting PdhS essential functions. Control immunoblots with anti-GFP antibodies revealed that this fusion protein was stable (data not shown). Observation using fluorescence microscopy showed that PdhS-CFP accumulated at one pole in more than 90% of the cells as previously described [17].

### Molecular techniques

DNA manipulations were performed according to standard techniques [28]. All plasmids used in this study (Table 1) were constructed by the Gateway™ technique (Invitrogen). To construct an *aidB *disruption mutant strain, a central 380-bp portion of the *aidB *CDS was amplified by PCR using AcoA and AcoB primers, and was subcloned into at the *Eco*RV site of pSK*oriTkan *vector [29]. The recombinant plasmid was transformed into the *E. coli *strain S17-1 and introduced into *B. abortus *544 Nal^R ^strain by mating. Clones in which the plasmid integrated in the genome were selected by growing the bacteria in the presence of kanamycin, and were checked by PCR using AcoDHP1 and pGEM-T-aval primers. Since *B. abortus *and *B. melitensis *are nearly identical at the genomic level, entry clones were recovered from the *B. melitensis *ORFeome version 1.1 [15]. LR recombination cloning procedure was performed as recommended by the manufacturer (Invitrogen Life-Technologies). The sequences of primers are available in Table 2.

**Table 2:** Primers

Name	Sequence 5' to 3'	Usage
*att*B1	ggggacaagtttgtacaaaaaagcaggct	Sequencing of coding sequence after ORFeome screening
*att*B2	ggggaccactttgtacaagaaagctgggt	Sequencing of coding sequence after ORFeome screening
acoDHP1	tagcaaatgcagtgcaag	PCR amplification for checking *aidB *disruption
pGEM-T-aval	ggaaacagctatgacca	PCR amplification for checking *aidB *disruption
AcoA	gcggcttacgggccataaa	Amplification of *B. abortus aidB *internal fragment
AcoB	gctgctcgaccaaaggcttg	Amplification of *B. abortus aidB *internal fragment

### Western blotting

For every fluorescent observations reported in this study, we carried out Western blot analyses with antibodies against YFP and CFP. These results allowed us to rule out the possibility that a particular localization pattern could result from protein degradation or from a deficiency in fusion protein production. Western blot analysis was carried out as described previously [8] with monoclonal antibodies against GFP (JL8, BD Biosciences) at 1/1000 dilution to check the stability of translational fusions to YFP or CFP.

### Microscopy

For fluorescence imaging, cell populations of *B. abortus *strains were immobilized on a microscope slide that was layered with a pad of 1% agarose containing phosphate-buffered saline (PBS) [30]. These slides were placed on a microscope stage at room temperature. Samples were observed on a Nikon i80 fluorescence microscope through a differential interference contrast (DIC, Normarski) 100X objective with a Hamamatsu Orca-ER LCD camera. Images acquisition and processing were done with NIS element (Nikon) software.

The detection of dead cells was performed with the Live/Dead BacLight kit L7007 (Invitrogen), according to manufacturer instructions.

### *Treatment of *B. abortus *strains with a DNA-alkylating agent*

*B. abortus *strains were grown in 2YT at 37°C overnight, centrifuged and the pellet was resuspended in PBS to a cell density of 10^9 ^c.f.u./ml (optical density of 0.33 at 600 nm). 500 μl of these cell suspensions were diluted into 5 ml of 2YT and exposed to methanesulphonic acid ethyl ester (EMS) at final concentrations of 0, 0.2, 0.4 and 1.0%. These suspensions were incubated at 37°C with shaking for 1 h or 4 h, and aliquots (1 ml) were recovered, washed once in PBS, and serially diluted in PBS. 100 μl of these cell suspensions were spread on individual 2YT agar plates. These plates were incubated for 72 h at 37°C, and the c.f.u. were enumerated.

### Cellular infection and immunofluorescence labelling

Infections and immunofluorescence of HeLa cells and RAW264.7 macrophages by the different *B. abortus *strains were performed as described previously [6]. *Anti-Brucella *lipopolysaccharide O-chain monoclonal antibody 12G12 [31] was used. The secondary antibody used was Texas red-conjugated anti-rabbit IgG (Molecular Probes) diluted 500 times.

## List of abbreviations

ACAD: Acyl-CoA dehydrogenase; BCV: *Brucella*-containing vacuole; CDS: coding sequence; CFP: cyan fluorescent protein; DIC: differential interference contrast; EMS: methanesulphonic acid ethyl ester; ER: endoplasmic reticulum; pCDS: predicted coding sequence; ROS: reactive oxygen species; YFP: yellow fluorescent protein

## Authors' contributions

DD made all experiments, except macrophages infections reported in Figure 2B, that were performed by CM. JJL and XDB participated to the design of the work. DD and XDB wrote the manuscript. All authors read and approved the final manuscript.

## Supplementary Material

Additional file 1 **Sequence alignment between *E. coli *and *B. abortus *AidB**. Alignment of *E. coli *and *B. abortus *AidB highlighting the conserved parts of these enzymes, and the absence of high similarity in the C-terminal portion of these proteins. Click here for file

Additional file 2 **3D structure of *E. coli *AidB and 3D model of *B. abortus *AidB**. The 3D model of *B. abortus *AidB suggests that while regions involved in tetramer formation are conserved, the C-terminal domain involved in DNA binding is not conserved. Click here for file

Additional file 3 **Infection of RAW264.7 macrophages with wild-type and *aidB *mutants strains**. c.f.u. countings during macrophages infection show that *aidB *mutation or overexpression does not dramatically impair intracellular survival and replication of *B. abortus*. Click here for file
