# Supplementary material for: The alkylation response protein AidB is localized at the new poles and constriction sites in Brucella abortus
Source: BMC Microbiol. 2011 Nov 23;11:257. doi: 10.1186/1471-2180-11-257 (PMC3236019; doi:10.1186/1471-2180-11-257)
Supplement: Additional file 1 — Sequence alignment between E. coli and B. abortus AidB. Alignment of E. coli and B. abortus AidB highlighting the conserved parts of these enzymes, and the absence of high similarity in the C-terminal portion of these proteins. [file 1471-2180-11-257-S1.DOC]

*E. coli* 4 QTHTVFNQPIPLNNSNLYLSDGALCEAVTREGAGWDSDFLASIGQQLGTAESLELGRLAN 63

+TH V NQ P+ +N YL D L + R ++ L G+ + +AE+ +L RLAN

*B. abortus* 11 KTHEVTNQTPPITGTNAYLGDPLLMQIAARFPKELHTE-LEQAGRFVLSAEAQDLARLAN 69

*E. coli* 64 VNPPELLRYDAQGRRLDDVRFHPAWHLLMQALCTNRVHNLAWEEDARSGA--FVARAARF 121

P+L +D QGRR+D V +HPA+H LM+ +H+ WE++ ARAARF

*B. abortus* 70 TELPKLRTHDRQGRRIDLVEYHPAYHALMRRSVAQGLHSSIWEDNPLESGRRHQARAARF 129

*E. coli* 122 MLHAQVEAGSLCPITMTFAATPLLLQMLPAPFQDWTTPLLSDRYDSHLLPGGQKRGLLIG 181

L AQ+EAG LCP+TMT A+ L+ P ++ W+ +LS +YD P +K+G+ +G

*B. abortus* 130 YLTAQLEAGHLCPLTMTSASLAALMAS-PEVYKQWSPAVLSRKYDFSQKPAFRKQGVTLG 188

*E. coli* 182 MGMTEKQGGSDVMSNTTRAERLEDGSYRLVGHKWFFSVPQSDAHLVLAQTAGGLSCFFVP 241

MGMTEKQGG+DV +N TRAE G++RL GHKWF S P SDA L LAQT GLSCF +P

*B. abortus* 189 MGMTEKQGGTDVRANATRAEPAIGGAWRLTGHKWFMSAPMSDAFLTLAQTKEGLSCFLLP 248

*E. coli* 242 RFLPDGQRNAIRLERLKDKLGNRSNASCEVEFQDAIGWLLGLEGEGIRLILKMGGMTRFD 301

R G+ N +RLKDKLGNRSNAS EVEF A+G ++G GEG++ I+ M +TR D

*B. abortus* 249 RLGEKGESNGFFFQRLKDKLGNRSNASSEVEFDGALGQMIGSPGEGVKTIMDMVTLTRLD 308

*E. coli* 302 CALGSHAMMRRAFSLAIYHAHQRHVFGNPLIQQPLMRHVLSRMALQLEGQTALLFRLARA 361

CA+ S +MR + A++H+ RHVFG PL++QPLM+ VL+ MAL + G TAL RLARA

*B. abortus* 309 CAVASAGLMRSGLAEAVHHSRHRHVFGKPLVEQPLMQRVLADMALDVAGATALSMRLARA 368

*E. coli* 362 WDRRA-DAKEALWARLFTPAAKFVICKRGMPFVAEAMEVLGGIGYCEESELPRLYREMPV 420

+D A D EA +AR TP K+ +CK + EAME LGG GY E+ L R YRE PV

*B. abortus* 369 FDMAASDRAEAAFARSMTPVVKYWVCKIAPALLYEAMECLGGNGYIEDGNLARAYREAPV 428

*E. coli* 421 NSIWEGSGNIMCLDVLRVLNKQAGVYDLLSEAFVEVKGQDRYFDRAVRRLQQQLRKPAEE 480

N+IWEGSGN+M LDV RVL++ ++D + + G V R QL + +

*B. abortus* 429 NAIWEGSGNVMALDVARVLSRAPALFDGVLDWISGQLGPRGQGTIDVLRAALQLTETDQG 488

*E. coli* 481 LGREITHQLFLLGCGAQMLKYASPPMAQAWCQVML 515

+ R +T QL A++ + + +A A+ + L

*B. abortus* 489 VARLLTEQLAFAAAAAELRQLGADDIADAFIETRL 523

**Additional file 1.** **Sequence alignment between *E. coli* and *B. abortus* AidB.** Non-similar residues at N-terminus and C-terminus are not indicated. Similar but not identical residues are indicated by "+".
