# Supplementary material for: The alkylation response protein AidB is localized at the new poles and constriction sites in Brucella abortus
Source: BMC Microbiol. 2011 Nov 23;11:257. doi: 10.1186/1471-2180-11-257 (PMC3236019; doi:10.1186/1471-2180-11-257)
Supplement: Additional file 2 — 3D structure of E. coli AidB and 3D model of B. abortus AidB. The 3D model of B. abortus AidB suggests that while regions involved in tetramer formation are conserved, the C-terminal domain involved in DNA binding is not conserved. [file 1471-2180-11-257-S2.DOC]

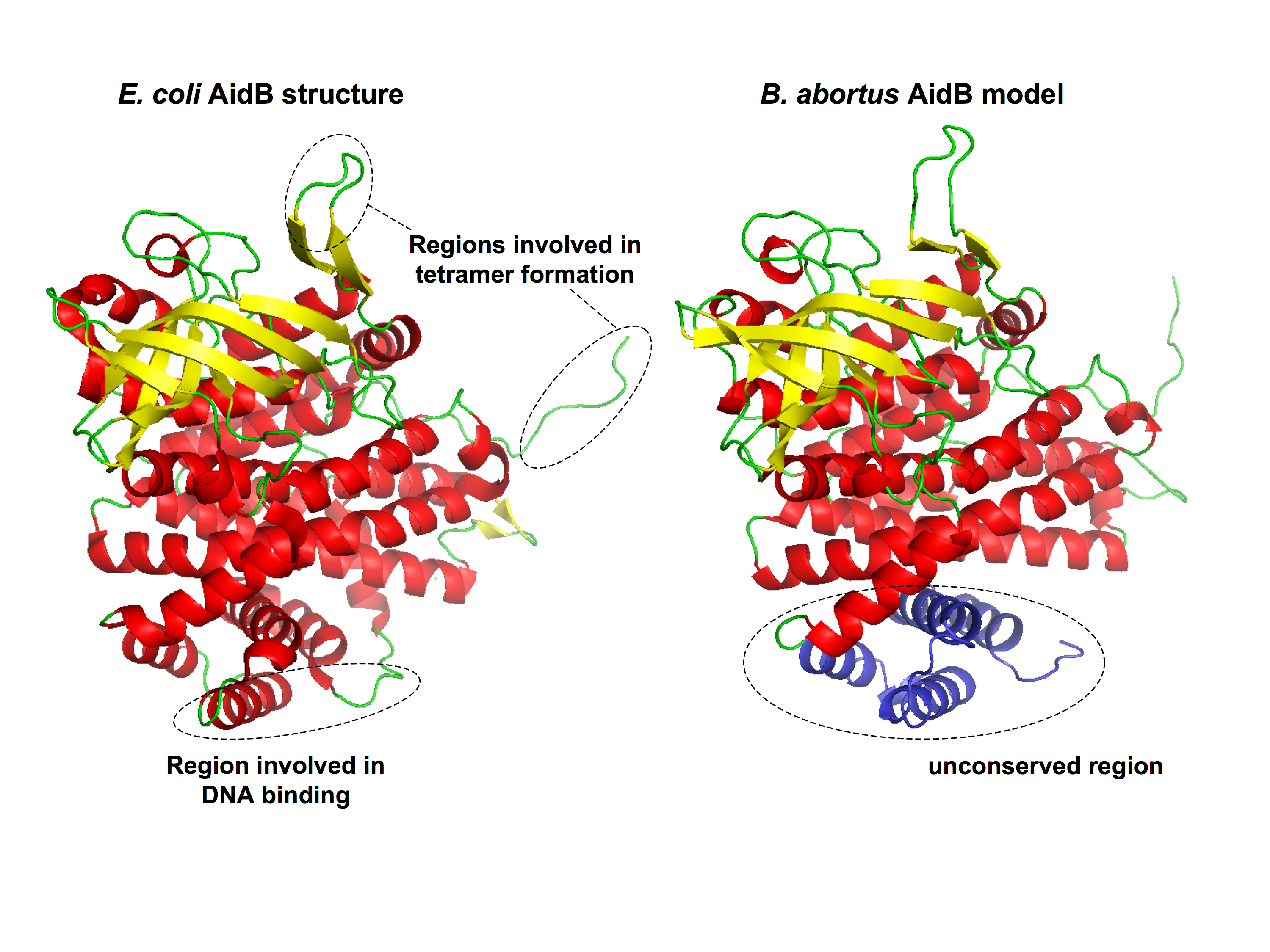


**Additional file 2.** **3D structure of *E. coli* AidB and 3D model of *B. abortus* AidB.** The 3D model was constructed using the Esypred modelling server (Lambert *et al.* *Bioinformatics* **18**, 1250). In the *E. coli* AidB structure, the regions involved in tetramer formation and DNA binding are indicated. The region corresponding to the DNA binding site is not conserved in *B. abortus* sequence (<15% sequence identity), suggesting that the DNA binding function may be absent in *B. abortus* AidB.
