# Supplementary material for: The alkylation response protein AidB is localized at the new poles and constriction sites in Brucella abortus
Source: BMC Microbiol. 2011 Nov 23;11:257. doi: 10.1186/1471-2180-11-257 (PMC3236019; doi:10.1186/1471-2180-11-257)
Supplement: Additional file 3 — Infection of RAW264.7 macrophages with wild-type and aidB mutants strains. c.f.u. countings during macrophages infection show that aidB mutation or overexpression does not dramatically impair intracellular survival and replication of B. abortus. [file 1471-2180-11-257-S3.DOC]

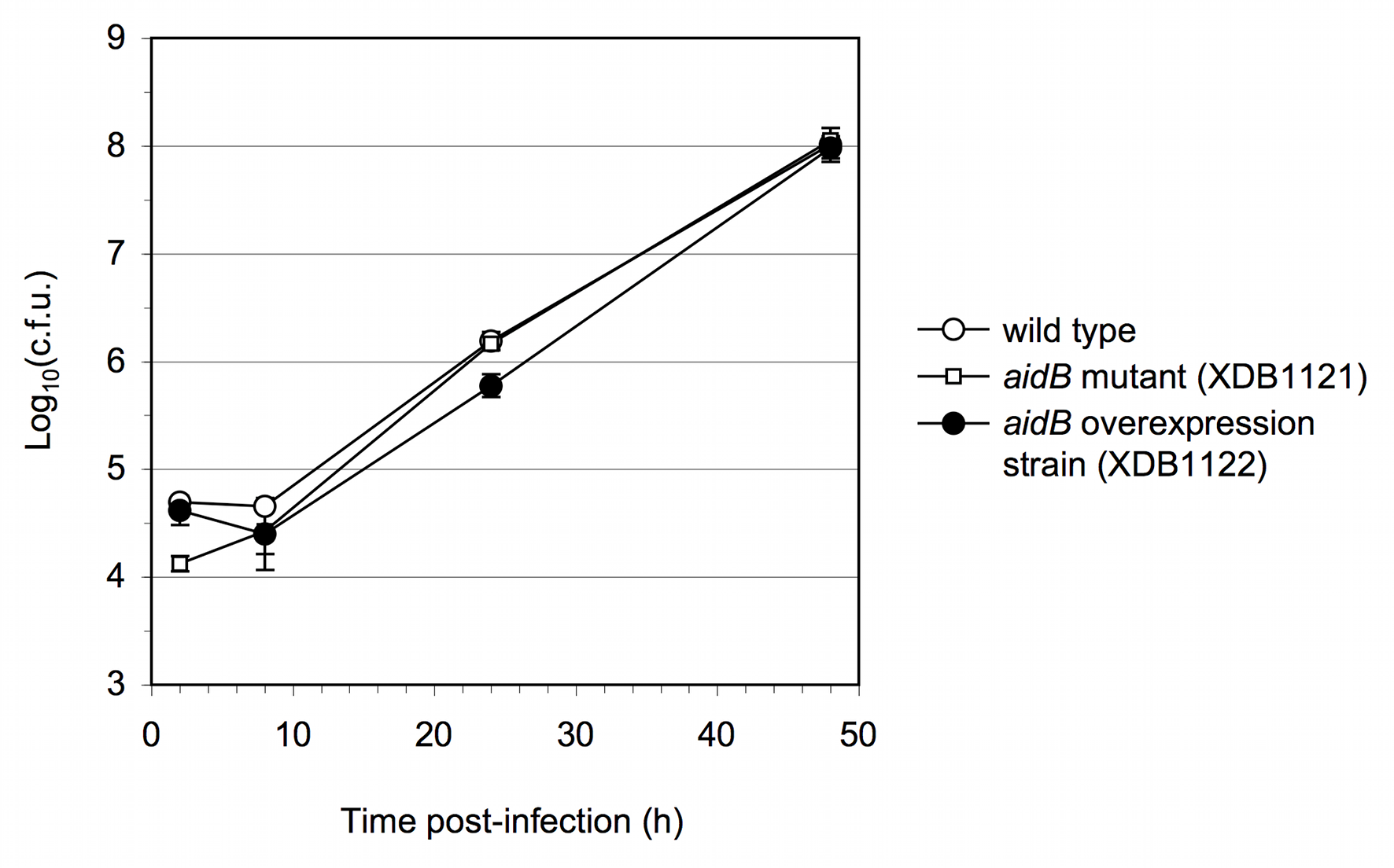


**Additional file 3.** **Infection of RAW264.7 macrophages with *B. abortus* wild type or *aidB* mutant strains.** RAW264.7 macrophages (2 10^5^ per well) were infected with a multiplicity of infection of 300 bacteria per cell. Intracellular bacterial colony forming units (c.f.u.) numbers were determined after lysis of infected cells as described previously (Delrue *et al.*, *Cell. Microbiol.* **3**, 487). Each determination was performed in duplicate and values are the means ± standard deviations from three independent experiments. The disruption or the overexpression of *aidB* do not dramatically affect the bacterial intracellular replication.
